# Suitable Fertilizer Application Depth Enhances the Efficient Utilization of Key Resources and Improves Crop Productivity in Rainfed Farmland on the Loess Plateau, China

**DOI:** 10.3389/fpls.2022.900352

**Published:** 2022-06-06

**Authors:** Guangzhou Chen, Tie Cai, Junying Wang, Yuhao Wang, Liangqi Ren, Peng Wu, Peng Zhang, Zhikuan Jia

**Affiliations:** ^1^College of Agronomy, Northwest A&F University, Yangling, China; ^2^Key Laboratory of Crop Physi-Ecology and Tillage Science in Northwestern Loess Plateau, Ministry of Agriculture and Rural Affairs, Northwest A&F University, Yangling, China; ^3^The Chinese Institute of Water-Saving Agriculture, Northwest A&F University, Yangling, China

**Keywords:** fertilizer application depth, root growth, aboveground growth, grain yield, resource use efficiency

## Abstract

Appropriate fertilizer application methods can help to improve crop yields. However, limited information is available regarding how different fertilizer application depths might affect crop production in dryland winter wheat-summer maize cropping in the Loess Plateau region of China. Therefore, we conducted field experiments in 2019–2020 and 2020–2021 to evaluate the effects of changing the fertilizer placement depth on summer maize (current crop) and winter wheat (succeeding crop) productivity, as well as the resource use efficiency and soil nitrate-nitrogen residue (SNR) level. Four fertilizer placement depths were tested comprising 5 cm (FD5), 15 cm (FD15), 25 cm (FD25), and 35 cm (FD35). The nitrogen uptake by summer maize in the two seasons was 10.0, 6.5, and 11.8% higher under FD15 compared with those under FD5, FD25, and FD35, respectively, because FD15 effectively increased the root length density, root surface area density, and rate of root bleeding sap. Due to the increased nitrogen uptake, the leaf area index, plant height, stem diameter, and accumulated dry matter were improved in summer maize. The interception of photosynthetically active radiation was 3.6, 3.7, and 5.9% higher under FD15 compared with those under FD5, FD25, and FD35, respectively. The summer maize grain yield increased by 13.9–22.4% under FD15 compared with the other treatments. In addition, the SNR in the deep soil (200–300 cm) was significantly lower under FD15 during the summer maize harvest (17.9–30.7%) compared with the other treatments. Moreover, FD15 increased the winter wheat (succeeding crop) grain yield (2.6–11.2%) and reduced the SNR in the 200–300 cm soil layer (8.8–16.8%) at the winter wheat harvest. The highest radiation use efficiency, precipitation use efficiency, and nitrogen use efficiency were obtained under FD15 in both summer maize and winter wheat. These results clearly suggest that depth fertilization of 15 cm enhanced the productivity and resource use efficiency for the current and subsequent crops in rainfed farmland in the Loess Plateau of China, as well as reducing the SNR in the deep soil to promote sustainable agricultural development. These findings provide a practical reference for optimizing fertilizer application management.

## Introduction

The Loess Plateau is a typical rainfed agricultural area in northern China (Qiang et al., 2022). The winter wheat–summer maize double cropping system is a key food production system in this region (Zhao et al., 2018) and it plays an important role in ensuring food security for China (Li et al., 2019). Over recent decades, the popularization and use of synthetic fertilizers have greatly contributed to enhanced food production, thereby alleviating hunger (Xia et al., 2017; Wang et al., 2020). However, the excessive pursuit of high yields and profits by farmers has led to the unreasonable use of chemical fertilizer (Shi et al., 2021; Qiang et al., 2022), thereby resulting in the waste of key production resources and severely damaging the ecological environment (Sainju et al., 2012; Zhang et al., 2019). Therefore, it is necessary to explore a fertilization strategy that can achieve a higher nitrogen use efficiency (NUE) and grain yield, but also minimize the consumption of natural resources and the deterioration of environmental conditions.

Recent studies have explored various fertilization management practices for improving crop productivity (Liu et al., 2015; Su et al., 2015; Gebre and Earl, 2021; Zhong et al., 2021). In particular, compared with broadcasting or mixing fertilizer, banding fertilizer in the soil can improve the NUE and crop yield in rice (Liu et al., 2015; Zhong et al., 2021), rape (Su et al., 2015), soybean (Gebre and Earl, 2021), wheat (Singh et al., 2005), and maize (Cheng et al., 2020; Wu et al., 2021). Therefore, banding fertilizer has some advantages for promoting crop production. These advantages are mainly due to banding fertilizer improving the availability of nutrients in the crop root zone to promote the growth and function of roots. Banding fertilizer in the root zone is an effective fertilization method and it has attracted much attention from researchers (Liu et al., 2015; Su et al., 2015; Gebre and Earl, 2021). Su et al. (2015) showed that fertilization at soil depths of 10 and 15 cm increased the taproot length and dry weight compared with depths of 0 and 5 cm. Moreover, recent field experiments have assessed the effects of deep-band fertilizer placement at different soil depths on soybean and they showed that the soybean root distribution and nutrient absorption varied significantly with the fertilizer placement depth (Gebre and Earl, 2021). However, the effects of the fertilization depth on the spatial structure of the roots in summer maize and the associated mechanism related to yield increases are still uncertain. Therefore, it is necessary to identify a suitable fertilization depth to further improve the fertilizer banding method.

The nitrogen absorbed by crop roots is an important element for the formation of chlorophyll and photosynthetic proteins in the leaves, and it is the key factor that ensures normal photosynthesis in crops (Poorter et al., 2010). Therefore, the growth status of the belowground crop parts (root system) is strongly related to the growth and developmental status of the aboveground parts (Wang et al., 2019). To a certain extent, the absorption of more nitrogen by crop roots is more conducive to the growth and development of aboveground organs (Ordonez et al., 2021). The aboveground morphological characteristics of plants (such as the plant height, leaf area, and stem diameter) determine the capacity of the crop canopy to intercept radiation, thereby affecting the ability of the crop to utilize radiation (Zhao et al., 2015; Wang et al., 2021). However, when plant growth is limited by nitrogen and water, the intercepted radiation will be reduced due to limited canopy expansion and the crop yield will decrease (Teixeira et al., 2014). Therefore, the efficient conversion of the key production resources (nitrogen, water, and radiation) into biomaterials such as grain will effectively enhance crop production, especially in high-altitude rainfed agricultural areas (Zhang et al., 2019).

The residual amount of nitrate-nitrogen in the soil (SNR) at the crop harvest is significantly affected by the fertilizer application method employed (Shi et al., 2021). If the fertilizer application method is unsuitable, nitrate-nitrogen applied to the soil cannot be absorbed and utilized by crops at the correct time (Hartmann et al., 2014). The nitrate-nitrogen will accumulate or migrate downward in the soil over time to increase the SNR at the crop harvest (Ju and Christie, 2011). Excessive SNR in deep soil cannot be absorbed and utilized by crops (Shi et al., 2021), and it also threatens the safety of groundwater (Delin and Stenberg, 2014). Wu et al. (2021) found that changing the fertilization method greatly affected the during SNR at the spring maize harvest. Therefore, we hypothesized that optimizing the fertilization depth may improve the SNR at the summer maize harvest and influence the growth of the subsequent crop (winter wheat).

Few previous studies have investigated the effects of different fertilization application depths in the summer maize season on the biomass, yield and resource use efficiency in the current season crop (summer maize), and the subsequent crop (winter wheat) (Yang et al., 2016; Cheng et al., 2020). Therefore, we conducted a 2-year experimental study in rainfed farmland in the Loess Plateau region of China. We tested four different fertilizer application depths in the summer maize season. The fertilizer application method in the winter wheat season was consistent with the local production practices. The aims of this study were: (1) to determine the effects of different fertilizer application depths on the crop root growth, canopy development, biomass accumulation, yield, and resource use efficiency in summer maize; (2) to compare and analyze the differences in the SNR at the summer maize harvest under different fertilizer application depths; and (3) to assess the effects of different fertilizer application depths in the summer maize season on the productivity and SNR at harvest for the subsequent crop (winter wheat). The results obtained in this study provide a theoretical basis and technical support for determining a suitable fertilization depth for dryland crop production in the Loess Plateau region of China. In addition, our results should help to improve crop yields and resource utilization efficiency.

## Materials and Methods

### Experimental Site Description

**FIGURE 1:**
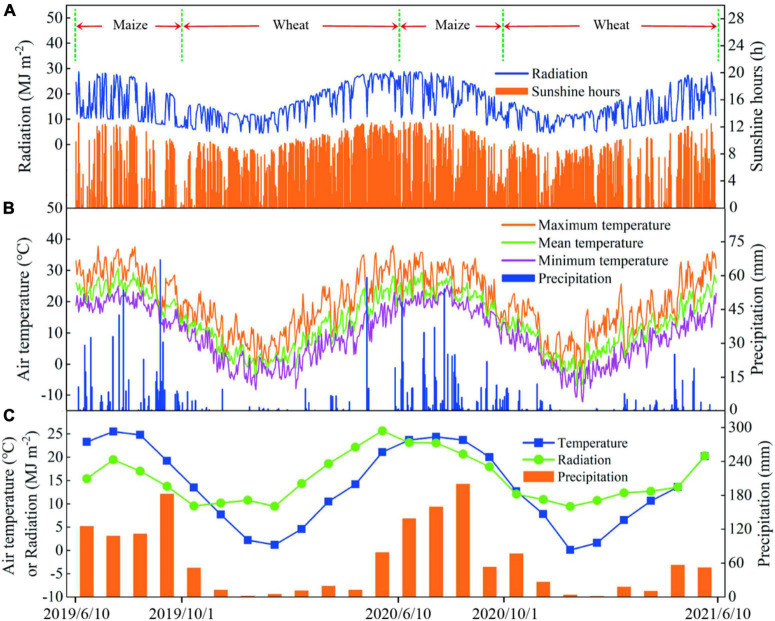
Daily solar radiation and sunshine hours from 2019 to 2021 **(A)**; daily temperature and precipitation **(B)**; monthly average daily temperature, radiation, and monthly cumulative precipitation **(C)**.

The experiment (2019–2021) was conducted at the Agricultural Experiment Station of Northwest A&F University located in Yangling, Shaanxi Province in northwestern China (34°29′N, 108°06′E; altitude 521 m) (Supplementary Figure 1A). The region has a semi-humid monsoon climate, but it is often prone to drought. In the past 30 years, the annual average precipitation was 600 mm (Supplementary Figure 1B) and the annual average temperature was 13.8°C (Supplementary Figure 1C). Daily meteorological data for the 2 years are shown in Figure 1. The total sunshine hours during 2019–2020 and 2020–2021 were 2,373.1 and 2,336.6 h, respectively, with precipitation of 721.3 and 775 mm, and total radiation of 5,669.2 and 5,656.7 MJ m^–2^. The meteorological data are from Yangling meteorological station (less than 500 m from the experimental field). The physical and chemical properties of the soil in the 0–60-cm layer at the test site are shown in Supplementary Table 1.

### Experimental Design and Field Management

**FIGURE 2:**
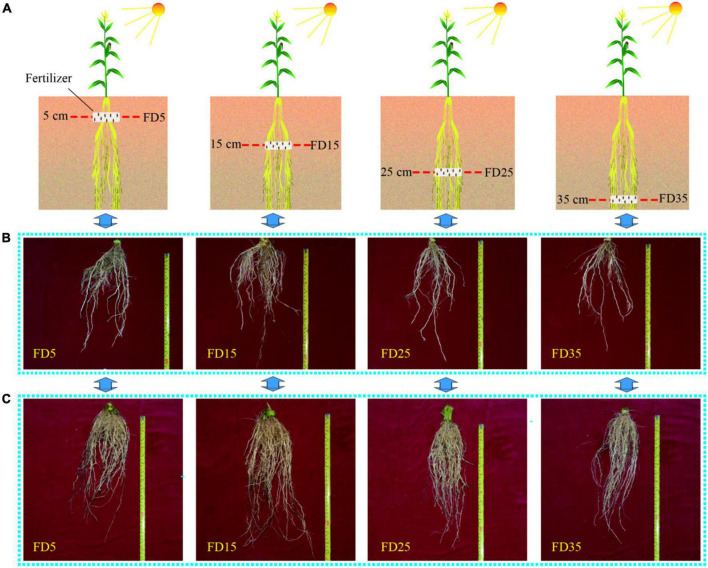
The schematic diagram of different fertilizer application depths **(A)**; maize root system in field soil profile at the six-leaf stage **(B)** and tasseling stage **(C)**. FD5, fertilizer application at a depth of 5 cm; FD15, fertilizer application at a depth of 15 cm; FD25, fertilizer application at a depth of 25 cm; and FD35, fertilizer application at a depth of 35 cm.

The experimental planting system comprised a wheat/maize rotation model. A randomized block trial design was used in this study. Four fertilizer application depths were set in the summer maize season: (1) FD5, N-P-K fertilizer banded placement at a soil depth of 5 cm, as the control treatment; (2) FD15, N-P-K fertilizer banded placement at a soil depth of 15 cm; (3) FD25, N-P-K fertilizer banded placement at a soil depth of 25 cm; and (4) FD35, N-P-K fertilizer banded placement at a soil depth of 35 cm, and three replicates were conducted for each treatment. The specific fertilizer application depth settings are shown in Figure 2A. The fertilizer application method in the winter wheat season was consistent with the actual production practices of local farmers.

Maize variety Zhengdan 958 (Liu et al., 2018; Meng et al., 2021) and wheat variety Xinong 979 (Ali et al., 2019; Xu et al., 2020), planted widely in local production, were selected in this experiment. Maize variety Zhengdan 958 (cultivated by Henan Academy of Agricultural Sciences) has the advantages of high yield, stable yield, disease resistance, and lodging resistance. It is the maize variety with the largest planting area in China. Wheat variety Xinong 979 (cultivated by Northwest A&F University) has the advantages of strong tillering ability and a high panicle rate. It is widely planted in winter wheat areas in northern China.

Before sowing the summer maize seeds, a subsoiling machine (Hebei nongha 1s-220, China) was used to uniformly subsoil the soil in all the test plots to a depth of 40 cm. Then, each test plot was manually trenched to the required soil depth (5, 15, 25, or 35 cm). The base fertilizers applied in the ditch comprised 170 kg of N ha^–1^ urea (46% N, Shaanxi Weihe Heavy Chemical Co., Ltd., China), 100 kg of P_2_O_5_ ha^–1^ calcium superphosphate (16% P_2_O_5_, Shaanxi Baoji Fenghe Chemical Co., Ltd., China), and 60 kg of K_2_O ha^–1^ potassium chloride (51% K_2_O, Russia), before backfilling with soil. Summer maize was manually sown at the beginning of June every year and the harvest occurred on September 30. The sowing depth of summer maize was about 5 cm. The planting density was 67,500 ha^–1^, the plant spacing was 24.7 cm, and the row spacing was 60 cm. Wheat was sown in the middle or late October every year. The row spacing of winter wheat was 20 cm, and the sowing amount was 180 kg hm^–2^. Wheat was harvested in early June of the following year. The amounts of nitrogen, phosphorus, and potassium fertilizers were 190 kg of N ha^–1^ urea (46% N), 100 kg of P_2_O_5_ ha^–1^ calcium superphosphate (16% P_2_O_5_), and 100 kg of K_2_O ha^–1^ potassium chloride (51% K_2_O), respectively. Each year, all fertilizers were broadcast evenly by hand over the soil surface on each plot as a basal fertilizer at sowing wheat before plowing immediately into the top 20-cm soil layer using a rotavator. No irrigation was applied during the experiment. During the experiment, pests and diseases were fully controlled by the application of chemicals. Weeds were controlled by manual hoeing. In addition, other common cropping management practices were consistent with that of local farmland.

### Sampling and Measurements

#### Root Length Density (RLD), Root Surface Area Sensity (RSD), and Rate of Root Bleeding Sap

In the six-leaf stage (V6), tasseling stage (VT), and grain-milking stage (R3) of summer maize, three representative plants were selected from each plot, and the roots were excavated by profile method (Supplementary Figure 2). According to Xu et al. (2021), the excavated roots were carefully cleaned. Then, a scanner (Epson V700, Indonesia) was used to obtain the root image file. Root images were analyzed to determine the RLD and RSD using WinRHIZO version 5.0 (Regent Instruments Inc., Quebec City, QC, Canada). The rate of root bleeding sap at the V6, VT, and R3 stages of summer maize was measured according to the method provided by Wang et al. (2012).

#### Leaf Area Index (LAI), Plant Height, and Stem Diameter

At the three-leaf stage (V3), V6, twelve leaf stage (V12), VT, R3, maturity stage (R6) for summer maize and the regreening stage, jointing stage, anthesis stage, filling stage, and maturity stage for winter wheat, the green leaf areas of the plants were measured using a tape measure with a minimum scale of 1 mm. The leaf area per maize or wheat plant (cm^2^) was calculated using the following equation (Birch et al., 2003):


(1) $$L\,A=\sum_{1}^{n}a_{i}\times b_{i}\times \text{c}$$

where *LA* (cm^2^) is the leaf area per plant, *ai* (cm) and *bi* (cm) are the midvein length and maximum width of the ith leaf, respectively, *n* is the number of leaves, and *c* is the conversion coefficient [0.75 for the maize according to Zhang et al. (2019a), 0.77 for the wheat according to Li et al. (2020)].

LAI (m^2^ ha^–1^) was calculated using the following equation (Zhang et al., 2019a).


$$L\,A\,I=l\,e\,a\,f\,a\,r\,e\,a\,\left(m^{2}\,p\,l\,a\,n\,t^{-1}\right)$$

(2) $$\times plantdensity\left(plantsha^{-1}\right)/10,000\left(m^{2}ha^{-1}\right)$$

At V3, V6, V12, VT, R3, and R6 for summer maize and the regreening stage, jointing stage, anthesis stage, filling stage, and maturity stage for winter wheat, the height of the plants in each experimental plot was measured using a tower ruler (Shaanxi Deruite Industry and Trade Co., Ltd., China) with a minimum scale of 1 mm. At the same time, the stem diameter of maize was measured using a tape measure.

#### Dry Matter Accumulation and Nitrogen Uptake

At the growth stages in V3, V6, V12, VT, R3, and R6 for summer maize or the regreening stage, jointing stage, anthesis stage, filling stage, and maturity stage for winter wheat, five maize plants with consistent growth or two rows of wheat plants with a length of 0.5 m were randomly selected in each experimental plot. The dry matter mass and nitrogen uptake of these plants were measured. Each plant was divided, dried in a constant temperature precision blast drying oven at 105°C (BPG-9240, Shanghai, China) for 1 h, and, then, dried in a constant temperature precision blast drying oven at 75°C for 72 h. The accumulated dry matter was determined by drying to constant weight and weighing. The plant nitrogen concentration of summer maize and winter wheat was analyzed and determined using the standard Kjeldahl Autoanalyzer (Kjeltec 8400; FOSS, Denmark) (Wang et al., 2020).

#### Canopy Light Transmittance (LT), Intercepted Photosynthetically Active Radiation (IPAR), and Radiation Use Efficiency (RUE)

During clear and cloudless weather in VT and R3 of summer maize, the photosynthetically active radiation in the maize canopy was measured using a canopy analyzer (AccuPAR LP-80, United States) from 9:00 to 11:00. Each plot was measured six times diagonally between the rows and the average value was determined. Measurements were obtained in three layers comprising the upper part of the canopy (20 cm above the top of the plant), the ear layer (at the female ear), and the bottom of the canopy (20 cm above the ground). The equation (Liu et al., 2018) used to calculate LT (%) is as follows:


(3) L⁢T=P⁢A⁢R/T⁢P⁢A⁢R×100%

where *LT* is the light transmittance of the canopy, *PAR* is the photosynthetically active radiation measured in the ear layer or bottom layer, and *TPAR* is the total photosynthetically active radiation in the upper part of the canopy.

The IPAR for the plant canopy (MJ m^–2^) and RUE (g MJ^–1^) were calculated according to Zhang et al. (2019b) as follows:


(4) $$I\,P\,A\,R=\sum 0.5\,R\,\left(1-e^{-k\,L\,A\,I}\right)$$

(5) $$R\,U\,E_{G\,Y}=G\,r\,a\,i\,n\,y\,i\,e\,l\,d/I\,P\,A\,R$$

(6) $$R\,U\,E_{B\,Y}=B\,i\,o\,m\,a\,s\,s\,y\,i\,e\,l\,d/I\,P\,A\,R$$

where *IPAR* is the amount of photosynthetically active radiation intercepted by the plant canopy (MJ m^–2^), *R* is the daily solar radiation (MJ m^–2^ d^–1^), *k* is the extinction coefficient (0.65 for maize or wheat) (Monteith, 1969; Kiniry et al., 1996), *LAI* is the leaf area index, *RUE*_GY_ is the radiation use efficiency based on grain yield (g MJ^–1^), and *RUE*_*BY*_ is the radiation use efficiency based on biomass yield (g MJ^–1^).

#### Soil Nitrate-Nitrogen Concentration (SNC) and Distribution

At V6, V12, VT, and R3 for summer maize or the regreening stage, jointing stage, anthesis stage, and filling stage for winter wheat, the SNC of 0–100-cm soil layer (soil samples were collected every 10 cm in 0–40-cm soil layer and every 20 cm in 40–100-cm soil layer) was measured according to the method of Wang et al. (2020). At the same time, during the harvest of summer maize and winter wheat, the SNC of the 0–300-cm soil layer was measured.

The residual nitrate-nitrogen (SNR, kg ha^–1^) in the 0–300-cm soil layer at harvest of maize or wheat was calculated with the following equation (Shi et al., 2021):


(7) $$S_{N\,R}=\sum_{i}^{300}\left(B\,i\times E\,i\times S_{N\,C-i}\times 10^{-1}\right)$$

Where, *Bi* (g cm^–3^) is the soil bulk density (ring knife method determination), *Ei* is the thickness of soil layer (cm), and *SNC-I* is the concentration of soil nitrate-nitrogen (mg kg^–1^) in different soil layers, i.e., *i* = 0– 10-, 10– 20-, 20– 30-, 30– 40-, 40– 60-,…, 280–300-cm soil layer.

#### Grain Yield, Precipitation Use Efficiency (PUE), and Nitrogen Use Efficiency (NUE)

Samples were collected at the maturity of summer maize and winter wheat. Five rows of maize (5 m length every harvest row) or three rows of wheat with a length of 1 m were randomly collected in each plot to measure grain yield.

The PUE (kg ha^–1^ mm^–1^) is calculated with the following equation (Zhang et al., 2021):


(8) P⁢U⁢E=G⁢r⁢a⁢i⁢n⁢y⁢i⁢e⁢l⁢d/P

Where *P* (mm) is the precipitation in the growing season of maize or wheat.

The calculation equation of NUE (kg kg^–1^) is as follows (Zhang et al., 2021):


(9) N⁢U⁢E=G⁢r⁢a⁢i⁢n⁢y⁢i⁢e⁢l⁢d/t⁢o⁢t⁢a⁢l⁢N⁢u⁢p⁢t⁢a⁢k⁢e

### Statistical Analysis

The SPSS 22.0 (SPSS Inc., Chicago, IL, United States) was used to analyze variance, the least significant difference test (*P* < 0.05), and correlation analysis to determine the differences and correlation degree in SNC, SNR, crop root traits, nitrogen uptake, canopy agronomic traits, IPAR, grain yield, RUE, PUE, and NUE. Canoco5.0 was used for redundancy analysis (RDA). ArcGIS 10.2 (Environmental Systems Research Institute, United States) was used to prepare geographic images. Origin 2021 (OriginLab, Northampton, MA, United States) was employed to make a histogram, scatter diagram, correlation diagram, etc.

## Results

### Summer Maize Root System

**FIGURE 3:**
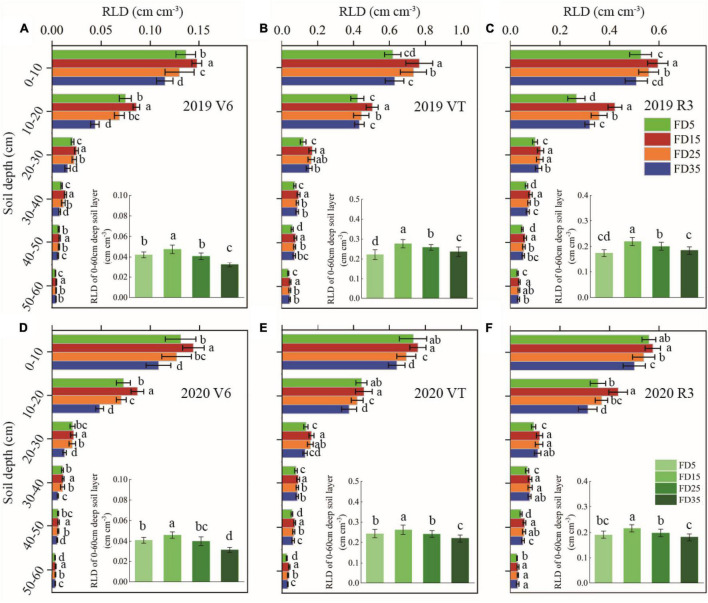
Effects of fertilizer application depths on root length density (RLD) at V6, VT, and R3 stages of summer maize in 2019 **(A–C)** and 2020 **(D–F)**. FD5, fertilizer application at a depth of 5 cm; FD15, fertilizer application at a depth of 15 cm; FD25, fertilizer application at a depth of 25 cm; and FD35, fertilizer application at a depth of 35 cm. The vertical bars stand for standard deviation. The different lowercase letters indicate a significant difference among the treatments at *P* < 0.05 using the LSD method.

**FIGURE 4:**
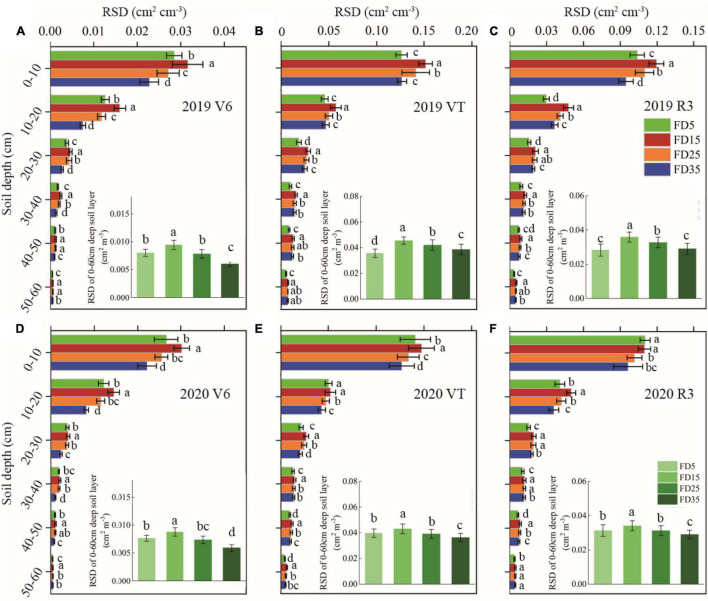
Effects of fertilizer application depths on root surface area density (RSD) at V6, VT, and R3 stages of summer maize in 2019 **(A–C)** and 2020 **(D–F)**. FD5, fertilizer application at a depth of 5 cm; FD15, fertilizer application at a depth of 15 cm; FD25, fertilizer application at a depth of 25 cm; and FD35, fertilizer application at a depth of 35 cm. The vertical bars stand for standard deviation. The different lowercase letters indicate a significant difference among the treatments at *P* < 0.05 using the LSD method.

The RLD of summer maize decreased gradually with the depth of the soil layer (Figure 3). The root system was mostly distributed in the 0–20-cm soil layer, followed by the 20–40-cm layer, and the 40–60-cm layer contained only a small amount of the root system (Figures 2–4). The spatial distribution of the RLD in the soil profile varied with the fertilizer application depth (Figure 3). In the two-summer maize growing seasons, compared with FD5, the RLD values in the 0–20-cm, 20–40-cm, and 40–60-cm soil layers were 16, 27, and 28.2% higher under FD15, respectively, 5.9, 21.9, and 20% higher under FD25, and −6, 11.7, and 14.7% higher under FD35, respectively. Therefore, FD15 and FD25 promoted the growth of the summer maize roots in all the soil layers. However, FD35 significantly reduced the RLD in the 0–20-cm soil layer for summer maize. The RSD values in the soil profiles exhibited similar trends to the RLD under all four treatments (Figure 4).

**FIGURE 5:**
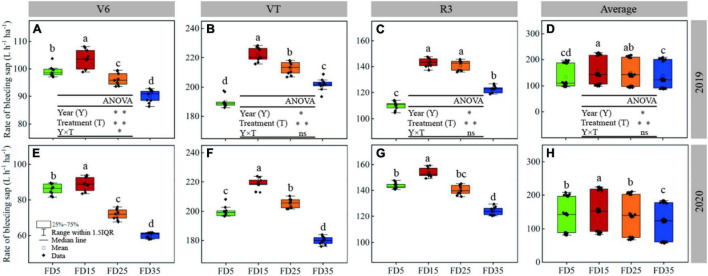
Effects of fertilizer application depths on rate of root bleeding sap at V6, VT, and R3 stages of summer maize in 2019 **(A–C)** and 2020 **(E–G)**; Effects of fertilizer application depths on the average rate of root bleeding sap of the whole summer maize growing season in 2019 **(D)** and 2020 **(H)**. FD5, fertilizer application at a depth of 5 cm; FD15, fertilizer application at a depth of 15 cm; FD25, fertilizer application at a depth of 25 cm; and FD35, fertilizer application at a depth of 35 cm. The different lowercase letters indicate a significant difference among the treatments at *P* < 0.05 using the LSD method. ns, no significant difference; *, significant at *P* < 0.05; ^**^, significant at *P* < 0.01.

During the summer maize growth period, the rate of root bleeding sap gradually increased under each treatment and reached a maximum in the VT stage, before decreasing in R3 (Figure 5). In the two-summer maize growing seasons, compared with FD5, the average rate of root bleeding sap was 12.3% higher under FD15 (*P* < 0.05), 4.7% higher under FD25 (*P* < 0.05), and 6% lower under FD35 (*P* < 0.05). Thus, the results showed that an appropriate fertilization depth (15 and 25 cm) promoted the rate of root bleeding sap in summer maize, whereas an excessive fertilization depth (35 cm) inhibited the rate of root bleeding sap.

### Leaf Area Index (LAI), Plant Height, and Stem Diameter in Summer Maize

**FIGURE 6:**
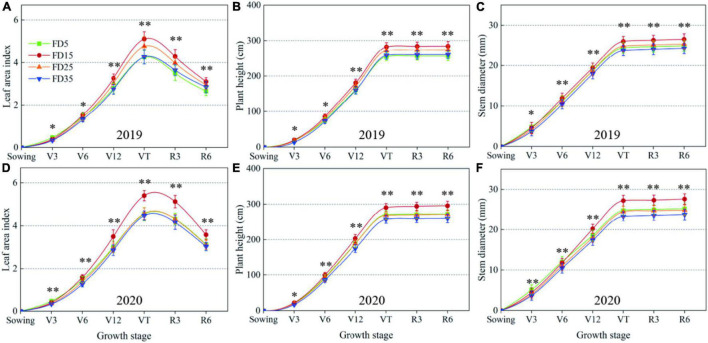
Effects of fertilizer application depths on leaf area index **(A,D)**, plant height **(B,E)** and stem diameter **(C,F)** of summer maize in 2019 and 2020. FD5, fertilizer application at a depth of 5 cm; FD15, fertilizer application at a depth of 15 cm; FD25, fertilizer application at a depth of 25 cm; and FD35, fertilizer application at a depth of 35 cm. The vertical bars stand for standard deviation. ns, no significant difference; *, significant at *P* < 0.05; ^**^, significant at *P* < 0.01.

The significant variations in the LAI started in the V6 stage, peaked in the VT stage, and then, gradually declined due to leaf senescence from the VT stage to the R6 stage (Figures 6A,D). On average, the LAI was 16.3% higher under FD15 than FD5 in the two-summer maize growing seasons. FD15 was highly effective but the LAI was significantly smaller than FD5 in the V3 stage (average decrease of 14.3%, *P* < 0.05). The LAI tended to be higher after the V12 stage under FD25 compared with FD5. However, the LAI in each growth stage was always smaller under FD35 than FD5. Therefore, the different fertilization depths affected the LAI, and, thus, the growth and development of summer maize.

In different summer maize growth stages, the plant height and stem diameter were significantly affected by the different fertilization depths (Figure 6). Under the different fertilization depth treatments, the differences in the summer maize plant height and stem diameter increased during the growth process. Over the two-summer maize growing seasons, the average plant height in the R6 stage was 9.4, 6.6, and 12.8% higher under FD15 than those under FD5, FD25, and FD35, respectively. Moreover, the average stem diameter was 8.1, 7.8, and 12.6% higher under FD15 than those under FD5, FD25, and FD35, respectively (*P* < 0.05). Therefore, FD15 effectively promoted the aboveground growth of summer maize to facilitate the establishment of a larger canopy.

### Nitrogen Uptake, Accumulated Dry Matter, and Intercepted Photosynthetically Active Radiation (IPAR) in Summer Maize

**FIGURE 7:**
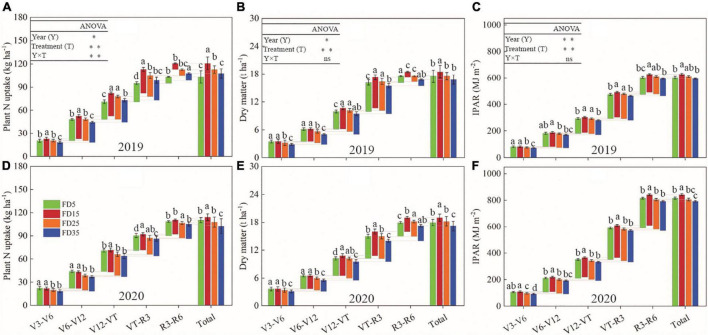
Effects of fertilizer application depths on plant nitrogen (N) uptake **(A,D)**, dry matter **(B,E)** and intercepted photosynthetically active radiation (IPAR, **C,F**) of summer maize in 2019 and 2020. FD5, fertilizer application at a depth of 5 cm; FD15, fertilizer application at a depth of 15 cm; FD25, fertilizer application at a depth of 25 cm; and FD35, fertilizer application at a depth of 35 cm. The vertical bars stand for standard deviation. The different lowercase letters indicate a significant difference among the treatments at *P* < 0.05 using the LSD method. ns, no significant difference; *, significant at *P* < 0.05; ^**^, significant at *P* < 0.01.

The cumulative nitrogen uptake by summer maize under different fertilization depths gradually increased during the maize growth and development process (Figures 7A,D). The nitrogen uptake amounts by summer maize under different fertilization depths were significantly higher in the V12–VT and VT–R3 periods compared with other periods. In the R6 stage over the 2 years, compared with FD5, the average cumulative nitrogen uptake amounts were 10% (*P* < 0.05) and 3.3% (*P* < 0.05) higher under FD15 and FD25, respectively, and 1.6% lower under FD35 (*P* > 0.05). The changes in the accumulated dry matter exhibited similar trends to those in the nitrogen uptake under all four treatments (Figure 7). In addition, the IPAR values in summer maize under different fertilization depths were significantly higher in V12–VT, VT–R3, and R3–R6 compared with V3–V6 and V6–V12 (Figures 7C,F). In V3–R6, compared with FD5, the 2-year average IPAR values were 3.6% higher under FD15, 0.1% lower under FD25, and 2.2% lower under FD35. Thus, FD15 enhanced the uptake of nitrogen and accumulated dry matter and IPAR in summer maize, whereas FD35 had the opposite effect.

### Canopy Transmittance (LT) and Radiation Use Efficiency (RUE) for Summer Maize

**FIGURE 8:**
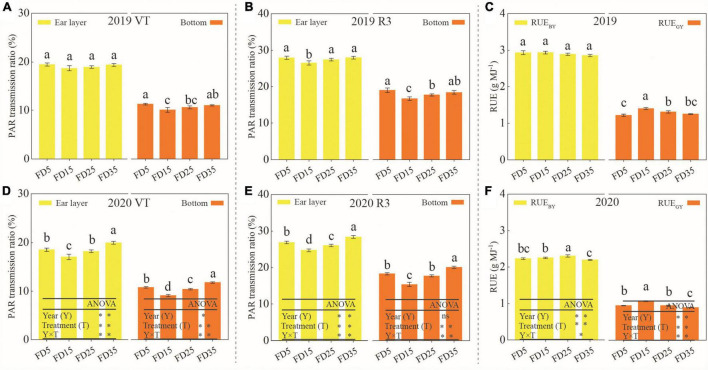
Effects of fertilizer application depths on photosynthetically active radiation (PAR) transmission ratio at VT **(A,D)** and R3 **(B,E)** stages of summer maize in 2019 and 2020. Effects of fertilizer application depths on radiation use efficiency (RUE) of summer maize in 2019 **(C)** and 2020 **(F)**. FD5, fertilizer application at a depth of 5 cm; FD15, fertilizer application at a depth of 15 cm; FD25, fertilizer application at a depth of 25 cm; FD35, fertilizer application at a depth of 35 cm; RUE_GY_, radiation use efficiency based on grain yield; and RUE_BY_, radiation use efficiency based on biomass yield. The vertical bars stand for standard deviation. The different lowercase letters indicate a significant difference among the treatments at *P* < 0.05 using the LSD method. ns, no significant difference; *, significant at *P* < 0.05; ^**^, significant at *P* < 0.01.

The different fertilizer application depths had significant effects on the LT for summer maize (Figures 8A,B,D,E). The LT for summer maize decreased from the canopy ear layer to the bottom layer. Over the two growing seasons, compared with FD5, the average LT values in the ear layer were 6.3 and 2.4% lower under FD15 and FD25, respectively, and the LT values in the bottom layer were 13.6 and 4.9% lower. Compared with FD5, the LT values in the ear layer were 3.1% higher under D35, and the LT values in the bottom layer were 3.3% higher. Moreover, the LT values in the ear layer and bottom layer were higher in R3 than VT under each treatment.

Over the two growing seasons, compared with FD5, the RUE_GY_ values were 14.1% higher under FD15 (*P* < 0.05), 4.1% higher under FD25 (*P* < 0.05), and 0.8% lower under FD35 (*P* > 0.05) (Figures 8C,F). The RUE_BY_ values did not differ significantly among FD5, FD15, FD25, and FD35 (*P* > 0.05), but compared with FD5, the values were 0.5 and 0.6% higher under FD15 and FD25, respectively, and 2.2% lower under FD35.

### Concentrations and Distributions of Nitrate-Nitrogen at Harvest of Summer Maize and Winter Wheat

**FIGURE 9:**
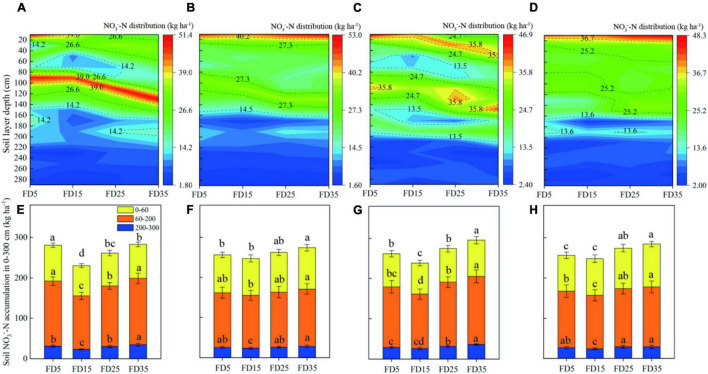
Nitrate distribution in the 0–300-cm soil layer at the harvest stage of summer maize and winter wheat. Soil nitrate distribution **(A,E)** at the 2019 summer maize harvest stage, **(B,F)** at the 2019–2020 winter wheat harvest stage, **(C,G)** at the 2020 summer maize harvest stage, and **(D,H)** at the 2020–2021 winter wheat harvest stage. FD5, fertilizer application at a depth of 5 cm; FD15, fertilizer application at a depth of 15 cm; FD25, fertilizer application at a depth of 25 cm; and FD35, fertilizer application at a depth of 35 cm. The vertical bars stand for standard deviation. The different lowercase letters indicate a significant difference among the treatments at *P* < 0.05 using the LSD method.

The differences in the SNC and SNR between treatments were greater during the summer maize harvest than in the winter wheat harvest (Figure 9 and Supplementary Figure 3). At the summer maize harvest in 2019, the differences in SNC and SNR in 0–300-cm soil layer followed the order of: FD35 > FD5 > FD25 > FD15, and at the summer maize harvest in 2020, they followed the order of: FD35 > FD25 > FD5 > FD15. FD15 reduced the SNR in the 0–300-cm soil layer (14%), while FD35 increased the SNR in the 0–300-cm soil layer (6.8%), compared with FD5. At the winter wheat harvests in 2019–2020 and 2020–2021, the differences in SNC and SNR in the 0–300-cm soil layer followed the order of: FD35 > FD25 > FD5 > FD15.

### Leaf Area Index (LAI), Plant Height, and Accumulated Dry Matter in Winter Wheat

**FIGURE 10:**
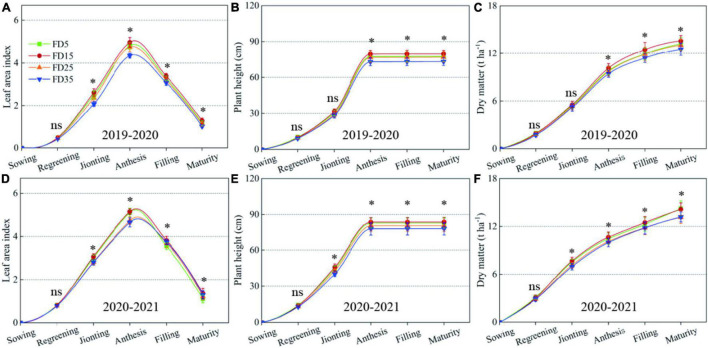
Leaf area index **(A,D)**, plant height **(B,E)** and dry matter **(C,F)** of winter wheat in 2019–2020 and 2020–2021. FD5, fertilizer application at a depth of 5 cm; FD15, fertilizer application at a depth of 15 cm; FD25, fertilizer application at a depth of 25 cm; and FD35, fertilizer application at a depth of 35 cm. The vertical bars stand for standard deviation. ns, no significant difference; *, significant at *P* < 0.05; ^**^, significant at *P* < 0.01.

The LAI under FD15 was slightly lower than that under FD5 in the regreening stage (0.2%, *P* > 0.05), but higher than those under FD5 in other growth stages, i.e., by 2–16.4% (Figures 10A,D). The LAI values under FD25 and FD35 were lower than those under FD5 in the regreening stage, jointing stage, and anthesis stage, but higher than those under FD5 in the filling and maturity. This showed the hat LAI of winter wheat was affected by both the depth of fertilization in the previous season and the growth stage of winter wheat.

At the maturity stage in the two-winter wheat growing seasons, the plant height was highest under FD15 (81.6 cm), and 1.9% (*P* > 0.05) higher than that under FD5 (Figures 10B,E). The plant heights under FD25 and FD35 were 2% (*P* > 0.05) and 5.8% (*P* < 0.05) lower than that under FD5, respectively.

On average over the 2 years, the accumulated dry matter amounts in winter wheat were significantly greater in the regreening-jointing and jointing-anthesis stages than in other periods (Figures 10C,F). In the R6 stage, compared with the 2-year average under FD5, the accumulated dry matter in wheat was 1.1% higher under FD15, but 4.6 and 7.4% lower under FD25 and FD35, respectively.

### Intercepted Photosynthetically Active Radiation (IPAR) and Radiation Use Efficiency (RUE) in Winter Wheat

**FIGURE 11:**
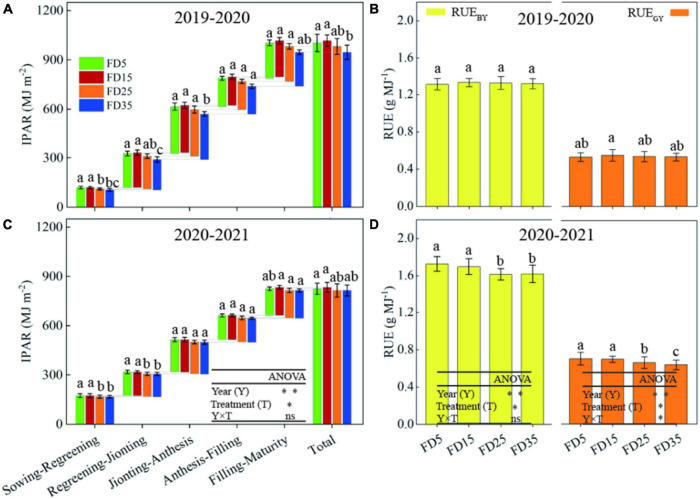
Winter wheat IPAR **(A,C)** and RUE **(B,D)** in 2019–2020 and 2020–2021. FD5, fertilizer application at a depth of 5 cm; FD15, fertilizer application at a depth of 15 cm; FD25, fertilizer application at a depth of 25 cm; FD35, fertilizer application at a depth of 35 cm; RUE_GY_, radiation use efficiency based on grain yield; and RUE_BY_, radiation use efficiency based on biomass yield. The vertical bars stand for standard deviation. The different lowercase letters indicate a significant difference among the treatments at *P* < 0.05 using the LSD method. ns, no significant difference; *, significant at *P* < 0.05; ^**^, significant at *P* < 0.01.

The accumulated IPAR in winter wheat gradually increased during the plant growth and development process (Figures 11A,C). Over the two growing seasons, compared with FD5, the average total IPAR was 1.2% higher under FD15, but 1.7 and 3.7% lower under FD25 and FD35, respectively.

Over the two growing seasons, compared with FD5, the RUE_GY_ value was 1.1% higher under FD15, but 2.9 and 5.2% lower under FD25 and FD35, respectively (Figures 11B,D). The RUE_BY_ values did not differ significantly among FD5, FD15, FD25, and FD35 (*P* > 0.05). Compared with FD5, the RUE_BY_ values were 3.3% and 3.4% lower under FD25 and FD35, respectively.

### Grain Yield, Precipitation Use Efficiency (PUE), and Nitrogen Use Efficiency (NUE) in Winter Wheat and Summer Maize

The different fertilizer application depths significantly affected the grain yields from summer maize (Table 1) and winter wheat (Table 2). On average, the summer maize grain yield was 18.1, 13.9, and 22.4% higher under FD15 compared with those under FD5, FD25, and FD35, respectively. The winter wheat grain yield was 2.6, 6.5, and 11.2% higher under FD15 than those under FD5, FD25, and FD35, respectively. The total annual grain yield was 11.5, 11.1, and 17.9% higher under FD15 than those under FD5, FD25, and FD35, respectively.

**TABLE 1:** Frequencies, means, and standard deviations for study variables.

	Whole sample		EHS group		Control group	
Characteristic	*n* (%)	M (SD)	*n* (%)	*M* (SD)	*n* (%)	*M* (SD)
Toddler age (in months) at the 14 month assessment		14.41 (1.28)		14.99 (1.20)		14.80 (1.28)
**Toddler sex**						
Females	78 (51%)		41 (52%)		37 (51%)	
Males	74 (49%)		38 (48%)		36 (49%)	
Toddler negative emotionality		3.00 (0.90)		2.97 (0.93)		3.02 (0.87)
Toddler productive vocabulary, 14 months		12.51 (11.22)		13.58 (11.97)		11.30 (10.27)
Toddler productive vocabulary, 24 months		53.04 (25.94)		57.30^+^ (24.98)		48.09 (26.35)
Maternal age (in years) at study enrollment		22.40 (5.07)		22.23 (5.04)		22.59 (5.13)
**Maternal race and ethnicity**						
White	105 (69%)		51 (65%)		54 (74%)	
Black	23 (15%)		13 (17%)		10 (14%)	
Latina	3 (2%)		1 (1%)		2 (3%)	
Other	6 (4%)		3 (4%)		3 (4%)	
Missing data	15 (10%)		11 (13%)		4 (5%)	
**Maternal education**						
< High school diploma	54 (35%)		27 (34%)		27 (37%)	
High school diploma	47 (31%)		26 (33%)		21 (29%)	
> High school diploma	33 (22%)		15 (19%)		18 (24%)	
Missing data	18 (12%)		11 (14%)		7 (10%)	
Annual family income in USD at study enrollment		9436.39 (7354.95)		8193.34 (6090.83)		10491.09^++^ (8176.13)
Parenting stress, clinical cutoff (>36)-14 months assessment	33 (22%)		18 (23%)		15 (21%)	
Parenting stress 14 months assessment		30.23 (9.52)		30.16 (10.04)		30.31 (8.99)
Proportion of appropriate MRC of all maternal comments made, 14 months		0.03 (0.03)		0.03 (0.03)		0.04 (0.04)
Proportion of appropriate MRC of all maternal comments made, 24 months		0.03 (0.03)		0.03 (0.03)		0.03 (0.03)
Proportion of MRC comments of all maternal comments made, 36 months		0.04 (0.02)		0.04 (0.02)		0.04 (0.02)
Total number of maternal comments at 14 months		96.38 (50.14)		103.39^+++^ (51.35)		88.78 (48.08)
Total number of maternal comments at 24 months		132.21 (50.83)		135.97 (50.58)		128.03 (51.18)
Total number of maternal comments at 36 months		128.90 (51.68)		130.41 (51.24)		127.02 (52.70)

*^+^p = 0.07; ^++^p = 0.09; ^+++^p = 0.11. There were no significant differences between EHS and control groups in demographic characteristics, parenting stress, child negative emotionality, or maternal mind-mindedness. Toddlers’ productive vocabulary was marginally (p = 0.07) greater for toddlers in the EHS group. Income was marginally (p = 0.09) higher for parents in the control group. The total number of parental comments at 14 months was marginally higher for parents in the EHS group (^++^p = 0.11).*

**TABLE 2:** Correlations among study variables.

Variables	1	2	3	4	5	6	7	8	9	10
1. Toddler age	−									
2. Toddler sex	0.11	−								
3. Toddler temperament	–0.08	–0.07	−							
4. Toddler productive language, 14 months	0.17*	0.07	–0.12	−						
5. Toddler productive language, 24 months	–0.04	0.12	–0.13	0.53***	−					
6. Maternal age	0.13	0.05	0.08	–0.06	–0.04	−				
7. Parenting stress, 14 months	–0.09	0.05	0.27***	–0.15	−0.15^†^	–0.00	−			
8. Maternal appropriate mind-related comments, 14 months	0.04	–0.08	–0.17	0.21*	0.31**	0.22*	–0.12	−		
9. Maternal appropriate mind-related comments, 24 months	0.00	0.02	–0.14	0.19*	0.11	0.16	–0.05	0.37***	−	
10. Maternal appropriate mind-related comments, 36 months	0.06	0.10	−0.18^†^	0.11	0.09	0.24*	–0.05	0.25*	0.36***	−

^†^p < 0.10; *p < 0.05; **p < 0.01; ***p < 0.001.

On average, PUE and NUE in summer maize were 18.1 and 7.4%, 13.8 and 7.1%, and 22.2 and 9.6% higher under FD15 compared with those under FD5, FD25, and FD35, respectively (Table 1). The PUE and NUE in winter wheat were 2.7 and 0.6%, 6.5 and 1.4%, and 11.2 and 1% higher under FD15 compared with those under FD5, FD25, and FD35, respectively (Table 2). The annual PUE and NUE were 11.6 and 5.6%, 11.1 and 5.1%, and 17.8 and 6.3% higher under FD15 compared with those under FD5, FD25, and FD35, respectively (Table 3).

**TABLE 3:** Annual grain yield, PUE, NUE, RUE_BY_, and RUE_GY_.

Treatment	Grain yield (kg ha^–1^)		PUE (kg ha^–1^ mm^–1^)		NUE (kg kg^–1^)		RUE_BY_ (g MJ^–1^)		RUE_GY_ (g MJ^–1^)	
	2019–2020	2020–2021	2019–2020	2020–2021	2019–2020	2020–2021	2019–2020	2020–2021	2019–2020	2020–2021
FD5	12656.4c	13563.3b	18.7c	18.7b	54.9ab	54.7b	1.92a	1.97a	0.79c	0.83b
FD15	14378.1a	14852.1a	21.2a	20.4a	57.0a	58.7a	1.95a	1.97a	0.87a	0.89a
FD25	13293.1b	13064.6bc	19.6b	18.0bc	55.8ab	54.5b	1.93a	1.95a	0.83b	0.81bc
FD35	12461.1c	12367.5d	18.4c	17.0d	54.6ab	54.4b	1.91a	1.90b	0.81bc	0.77d
**Significance level**										
Year (Y)	ns		**		*		*		ns	
Treatment (T)	**	**	**	**	ns	*	ns	*	**	**
Y × T	ns		*		ns		ns		ns	

PUE, precipitation use efficiency; NUE, nitrogen use efficiency; RUE_GY_, radiation use efficiency based on grain yield; and RUE_BY_, radiation use efficiency based on biomass yield. FD5, fertilizer application at a depth of 5 cm; FD15, fertilizer application at a depth of 15 cm; FD25, fertilizer application at a depth of 25 cm; and FD35, fertilizer application at a depth of 35 cm. Values within a column followed by the different lowercase letters indicate a significant difference at P < 0.05 using the LSD method. ns, no significant difference; *, significant at P < 0.05; **, significant at P < 0.01.

### Redundancy Analysis (RDA) and Correlation Analysis

**FIGURE 12:**
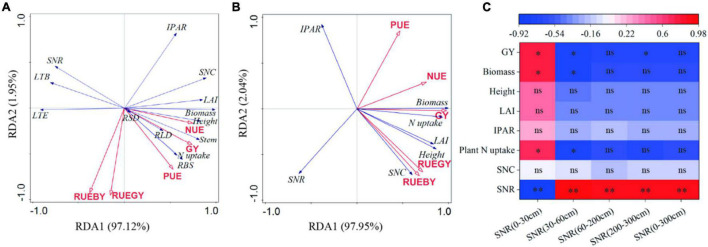
Redundancy analysis (RDA) based on the grain yield (GY), nitrogen use efficiency (NUE), precipitation use efficiency (PUE), radiation use efficiency based on grain yield (RUE_GY_), and radiation use efficiency based on biomass yield (RUE_BY_) of summer maize **(A)** and winter wheat **(B)**. Correlation analysis between indexes of winter wheat season and nitrate nitrogen residue (SNR) at harvest of previous crop (summer maize) **(C)**. SNC, soil nitrate nitrogen concentration; LAI, leaf area index; IPAR, intercepted photosynthetically active radiation; RLD, root length density; RSD, root surface area density; LTB, light transmittance at bottom; LTE, light transmittance at ear layer; RBS, rate of root bleeding sap. ns, no significant correlation; *, significant at *P* < 0.05; ^**^, significant at *P* < 0.01.

The RDA ordination plots were prepared to represent the relationships between the grain yield, PUE, NUE, RUE, crop growth index, SNC, and SNR (Figures 12A,B). The results showed that the grain yield, PUE, NUE, and RUE were positively correlated. The grain yield was positively correlated with all the indexes, except for SNR, LT, and IPAR.

Analysis based on the Pearson correlation coefficients (Figure 12C) showed that the SNR in the 0–30 cm soil layer at the summer maize harvest was positively correlated with the grain yield, biomass, plant height, LAI, IPAR, and nitrogen uptake by winter wheat, and highly significantly negatively correlated with the SNR in 0–300-cm soil layer at the winter wheat harvest (*P* < 0.01). The SNR values in the 30–60-cm, 60–200-cm, 200–300-cm, and 0–300-cm layers at the summer maize harvest were negatively correlated with various indexes in winter wheat, but significantly positively correlated with the SNR in the 0–300-cm soil layer at the winter wheat harvest (*P* < 0.01).

## Discussion

### Soil Nitrate-Nitrogen Concentration (SNC)

The SNC in the crop growing season is considered to be one of the most critical factors for regulating plant growth and development (Wang et al., 2020; Meng et al., 2021; Shi et al., 2021), especially in dryland agricultural production (Zhou et al., 2016; Muschietti-Piana et al., 2018; Wu et al., 2021). Optimizing the fertilization method can improve the SNC (Shi et al., 2021). In this study, compared with other fertilizer application depths, FD5 significantly decreased the SNC in the 0–40-cm soil layer in the summer maize growing season (Supplementary Figures 3A,E). The shallow fertilizer application depth (5 cm) may have resulted in the rapid decomposition of urea in the early stage to increase the gaseous losses of nitrogen and reduce the SNC (Liu et al., 2015). Liu et al. (2015) suggested that using an appropriate fertilizer application depth could prevent this problem. In the present study, when the fertilization depth increased from 5 to 15 cm, the SNC in the 0–40-cm soil layer increased significantly in the summer maize season (Supplementary Figures 3A,E). In addition, we found that the SNC decreased when the fertilization depth was 35 cm (Supplementary Figures 3A,E), possibly because the nitrogen applied to the deep soil (35 cm) could not be absorbed by the root system at the correct time, and the highly mobile NO3– migrated downward out of the root layer due to the occurrence of precipitation events (Figure 1 and Supplementary Figure 3). Therefore, we suggest that excessively shallow and deep fertilization depths were not conducive to the retention of nitrate-nitrogen in the root-soil.

Meng et al. (2021) found that optimizing the fertilization rate in the summer maize season enhanced the SNC in the winter wheat season (subsequent crop). Qiang et al. (2022) suggested that optimizing the fertilizer application depth in the summer maize season may have a similar effect and our results confirmed this hypothesis. We found that changing the fertilizer application depth in the summer maize season affected the SNC in the root layer in the winter wheat season (Supplementary Figure 3 and Figure 12C), possibly because the different fertilization depths affected the SNR distribution at the summer maize harvest (Qiang et al., 2022), thereby influencing the SNC in the winter wheat season (Meng et al., 2021). Therefore, we suggest that optimizing the fertilization depth in the summer maize season may improve the SNC in the winter wheat season and affect the growth and yield of winter wheat.

### Crop Growth, Nitrogen Uptake, and Intercepted Photosynthetically Active Radiation (IPAR)

Improving the SNC usually promotes plant root growth (Liu et al., 2015; Su et al., 2015). We found that the RLD, RSD, and rate of root bleeding sap were significantly higher under FD15 than those under FD5, FD25, and FD35 (*P* < 0.05), and Cheng et al. (2020) obtained similar results, probably because FD15 increased the SNC in the 0–40-cm soil layer compared with the other fertilizer application depths (Supplementary Figures 3A,E; Cheng et al., 2020). The RDA results also supported this explanation (Figure 12A). In addition, root growth and nitrogen absorption ability had important effects on the aboveground parts (LAI, plant height, and stem diameter) (Efthimiadou et al., 2010; Liu et al., 2018; Zhang et al., 2019a). The increased accumulated dry matter with an appropriate fertilizer application depth may have been related to the enhanced LAI, plant height, and stem diameter (Li et al., 2013; Yang et al., 2016). When we increased the fertilizer application depth from 5 to 15 cm, the summer maize biomass increased greatly (5.8%). However, when the fertilizer application depth was increased further, the summer maize biomass did not increase under FD25, and it decreased under FD35 (by 4.3%). Wu et al. (2021) also obtained similar results. Moreover, Yang et al. (2016) suggested that the fertilization depth should not be excessive for summer maize because the crop biomass was lower when the fertilizer application depth exceeded 24 cm. We consider that the fertilization depth of 15 cm significantly improved the nitrogen absorption capacity by summer maize roots (Figures 5, 7), promoted aboveground growth (Figure 6), and enhanced the IPAR by the canopy (Figures 7C,F). However, FD35 may have excessively restricted the availability of nutrients to hinder plant growth and development in the early growth stage (V3 and V6) (Figure 6) due to the reduced ability of plants to absorb nitrogen and intercept radiation (Figures 7, 8). Therefore, FD35 reduced the summer maize biomass compared with FD5.

In the wheat/maize rotation system, applying different fertilization methods to the current crop may affect the growth of the subsequent crop to some extent (Wang et al., 2020; Shi et al., 2021). The correlation analysis results showed that the biomass, plant height, LAI, IPAR, and nitrogen uptake in wheat were positively correlated with the SNR in the 0–30-cm soil layer at the maize harvest (Figure 12C). In particular, the biomass and nitrogen uptake in wheat were significantly positively correlated with the SNR in the 0–30-cm soil layer at the maize harvest (*P* < 0.05). Therefore, using different fertilizer application depths in the summer maize season affected the crop growth, nitrogen absorption, and IPAR in the winter wheat season (subsequent crop).

### Yield and Resources (Radiation, Water, and Nitrogen) Use Efficiency

In recent years, the global population has increased rapidly and the human demand for food has increased consequently. In addition, shortages of agricultural production resources are threatening the sustainable development of modern agriculture (Zhang et al., 2019b). Optimizing fertilization methods to promote the conversion of key production resources, such as radiation, water, and nitrogen, into grain may be effective for ensuring food security (Zhang et al., 2019b). Our results showed that compared with the other fertilizer application depths, the grain yield was 13.9–22.4% higher under FD15, and Cheng et al. (2020) obtained similar results. A fertilizer application depth of 15 cm was conducive to supplying nitrogen in the summer maize growing season to promote plant growth and yield formation (Yang et al., 2016; Cheng et al., 2020). Cheng et al. (2020) found that a fertilization depth of 15 cm significantly improved the crop NUE. We also found that compared with other fertilization depths, the PUE values were 13.8–22.2% higher and the RUE_GY_ values were 9.6–15.1% higher under FD15. Therefore, we suggest that a fertilization depth of 15 cm can improve the grain yield in summer maize and promote the effective utilization of key resources.

The correlation analysis results showed that the wheat grain yield was significantly positively correlated with the SNR in the 0–30-cm soil layer at the maize harvest (*P* < 0.05, Figure 12C). Thus, changing the fertilization depth in the summer maize season could have affected the winter wheat yield. Meng et al. (2021) found that optimizing the fertilization method in the summer maize season improved the summer maize yield, but also increased the winter wheat yield, and similar findings were obtained in our study. In the two-winter wheat growing seasons, compared with FD5, FD25, and FD35, the grain yield was 2.6–11.2% higher, PUE was 2.7–11.2% higher, NUE was 0.6–1.4% higher, and RUE_GY_ was 1.1–6.7% higher under FD15, probably because FD15 improved the SNC in the 0–30-cm soil layer in the wheat-growing season (Figure 12C and Supplementary Figure 3), promoted the uptake of nitrogen by plants (Table 2), and increased IPAR (Figure 11) to increase the grain yield and resource use efficiency (Table 2).

Liu et al. (2022) found that moderately deep banding with phosphorus could improve the wheat yield, phosphorus uptake, and phosphorus use efficiency. However, to the best of our knowledge, it is difficult to apply phosphorus fertilizer and other fertilizers (such as nitrogen fertilizer and potassium fertilizer) separately using different fertilization methods in actual production (Wu et al., 2021). However, the findings obtained by Liu et al. (2022) highlight some shortcomings of our research. Thus, in future research, we suggest that it may be useful to analyze how applying phosphorus and nitrogen at different depths might affect crop production.

### Soil Nitrate-Nitrogen Residue (SNR) at Crop Harvest

It is generally agreed that the main reason for the difference in SNR under different fertilizer application methods is that the amounts of nitrate-nitrogen absorbed by crops from the soil are significantly different (Zhang et al., 2015; Dai et al., 2016; Wang et al., 2020; Shi et al., 2021). We found that the average SNR in the 0–200-cm soil layer during the summer maize harvest followed the order of: FD35 > FD5 > FD25 > FD15 (Figures 9A,C,E,G). FD15 may have increased the uptake of nitrogen by plants (Figures 7A,D) to reduce the SNR in the 0–200-cm soil layer, whereas the opposite occurred under FD35 (Qiang et al., 2022), and the results obtained by RDA supported this explanation (Figure 12A). Hartmann et al. (2014) suggested that inappropriate fertilization methods will prevent nitrate-nitrogen from being absorbed by plants at the correct time, thereby increasing the SNR in the deep soil. We found that FD35 significantly increased the SNR in the 200–300-cm soil layer at the maize harvest compared with FD5 and FD15, possibly because applying the fertilizer at a depth of 35 cm distributed the soil nitrate-nitrogen mainly in the 30–40-cm soil layer during the crop growing season (Supplementary Figures 3A,E). Thus, the short roots in the early stage of crop growth could not absorb nitrate-nitrogen from the soil (Figure 7), so the nitrate-nitrogen leached down into the deep soil under the action of precipitation (Figures 9E,G). However, Miao et al. (2015) suggested that few crop roots can absorb nitrate nitrogen from the 200–300-cm soil layer. Therefore, we do not recommend applying fertilizer in deep soil layers, especially at depths of 35 cm or below.

The crop growth status significantly affects the SNR level at the harvest (Meng et al., 2021; Shi et al., 2021). RDA showed that the growth of winter wheat was negatively correlated with the SNR at the wheat harvest (Figure 12B), which is consistent with the results obtained in most previous fertilizer trials (Zhang et al., 2015; Wang et al., 2020). In addition, our results showed that compared with FD35, FD15 significantly reduced the SNR, especially in the 200–300-cm layer (Figures 9B,D,F,H), probably because FD15 promoted winter wheat growth and development to increase the amount of nitrogen absorbed by the crop (Table 2), thereby reducing the SNR at the harvest (Shi et al., 2021). Compared with the SNR in the 200–300-cm soil layer at the summer maize harvest, the SNR was significantly lower at the winter wheat harvest (13.1%, Figure 9). Shi et al. (2021) also obtained similar results and suggested that the migration of nitrate-nitrogen from the surface soil into deeper soil was consistent with the rainfall during the crop growing season. In the present study, the precipitation during the winter wheat growing season (October to June of the next year) accounted for only 25% of the annual precipitation, and the precipitation during the summer maize growing season (June to September) accounted for 68% (Figures 1B,C). Therefore, the SNR in the deep soil layer was higher during the summer maize harvest.

## Conclusion

The results obtained in this study showed that suitable fertilizer application depth (15-cm depth) promoted the nitrogen uptake of summer maize. This nitrogen uptake increase was essentially attributed to the improvement of nitrate-nitrogen concentration in root layer soil by suitable depth fertilization, which promoted the root growth and rate of root bleeding sap of summer maize. Suitable depth fertilization promoted plant height, stem diameter, and LAI of summer maize, thus increasing the ability of plants to intercept radiation. At the same time, suitable depth fertilization improved the utilization and transformation ability of summer maize to nitrogen, radiation, and precipitation. Therefore, these alterations constitute the key mechanisms underlying the increase in yield and resource use efficiency under suitable depth fertilization. Consistent with our hypothesis, optimizing the fertilizer application depth the of summer maize season promoted the growth of the succeeding season crop (winter wheat). In addition, optimizing the fertilizer application in the depth of summer maize season reduced the SNR of deep soil (200–300 cm) during the summer maize and winter wheat harvest. Therefore, optimizing fertilizer application depth via improved high plant yield and resource use efficiency will be more beneficial to the sustainable development of rainfed agriculture. The results of our experiment provide an effective fertilization strategy to help improve yield, alleviate agricultural resource shortages, and contribute to reducing the environmental pollution.

## Data Availability Statement

The original contributions presented in the study are included in the article/Supplementary Material, further inquiries can be directed to the corresponding authors.

## Author Contributions

GC, TC, PZ, and ZJ conceived and designed the experiments. GC, JW, YW, PW, and LR performed the experiments. GC analyzed the data and wrote the manuscript. GC, PZ, and TC reviewed and revised the manuscript and corrected the English language. All authors reviewed and approved the manuscript for publication.

## Conflict of Interest

The authors declare that the research was conducted in the absence of any commercial or financial relationships that could be construed as a potential conflict of interest. The reviewer LD declared a shared affiliation with the authors to the handling editor at the time of review.

## Publisher’s Note

All claims expressed in this article are solely those of the authors and do not necessarily represent those of their affiliated organizations, or those of the publisher, the editors and the reviewers. Any product that may be evaluated in this article, or claim that may be made by its manufacturer, is not guaranteed or endorsed by the publisher.
