# Supplementary material for: Suitable Fertilizer Application Depth Enhances the Efficient Utilization of Key Resources and Improves Crop Productivity in Rainfed Farmland on the Loess Plateau, China
Source: Front Plant Sci. 2022 Jun 6;13:900352. doi: 10.3389/fpls.2022.900352 (PMC9208331; doi:10.3389/fpls.2022.900352)
Supplement: Supplementary file 1 [file Table_1.doc]

***Supplementary Material***

**1. Supplementary Sampling and measurements**

The daily solar radiation required in this study was estimated based on the daily sunshine hours at Yangling meteorological station by referring to the international Angstrom empirical formula (Pickering et al., 1994). The formula is as follows:

$\text{R}=\left(as+bs\times h/H\right)\times Ra$,

where *R* is the total solar radiation (MJ m^–2^), *as* and *bs* are empirical coefficients related to the local air quality (i.e., *as* and *bs* are generally 0.25 and 0.5, respectively, in the Loess Plateau region; Zhang et al., 2019), *H* denotes the maximum sunshine hours (h), *h* is the measured average actual sunshine time (h), and *Ra* is the astronomical radiation (i.e., solar radiation on sunny days, MJ m^–2^).

The astronomical radiation (Ra) is calculated as follows:

$\text{Ra}=37.586\times\text{d}\times\left(\psi\sin\phi\sin\delta+\cos\phi\cos\delta\sin\psi\right)$,

where *d* is the relative distance between the Earth and the Sun, *δ* is the declination (°), and *ψ* is the angle of sunset (°), which are calculated as follows:

$$\text{d}=1+0.033\times\cos\left(2\pi/365\times J\right)$$

$$\delta=0.4093\times\text{sin}\left(2\pi\text{/}365\times\text{J-}1.045\right)$$

$\psi=\text{arccos}\left(\text{-tan}\phi\tan\delta\right)$,

where the parameter *J* represents the day of the year, e.g., January 1 is the first day of the year.

1. **Supplementary Tables and Figures**

**Table S1** The physio-chemical properties of the soil before the experiment at the site.

| Soil layer (cm) | NO_3_^-^-N (mg kg^-1^) | NH_4_^+^-N (mg kg^-1)^ | Total N (g kg^-1)^ | Available P  (mg kg^-1^) | Available K (mg kg^-1)^ | Bulk density (g cm^-3^) | Organic carbon  (g kg^-1^) |
| --- | --- | --- | --- | --- | --- | --- | --- |
| 0-20 | 21.52 | 6.16 | 0.95 | 14.64 | 132.7 | 1.42 | 11.32 |
| 20-40 | 8.14 | 5.83 | 0.82 | 5.25 | 115.1 | 1.47 | 10.14 |
| 40-60 | 4.88 | 5.42 | 0.61 | 3.79 | 94.6 | 1.44 | 9.08 |


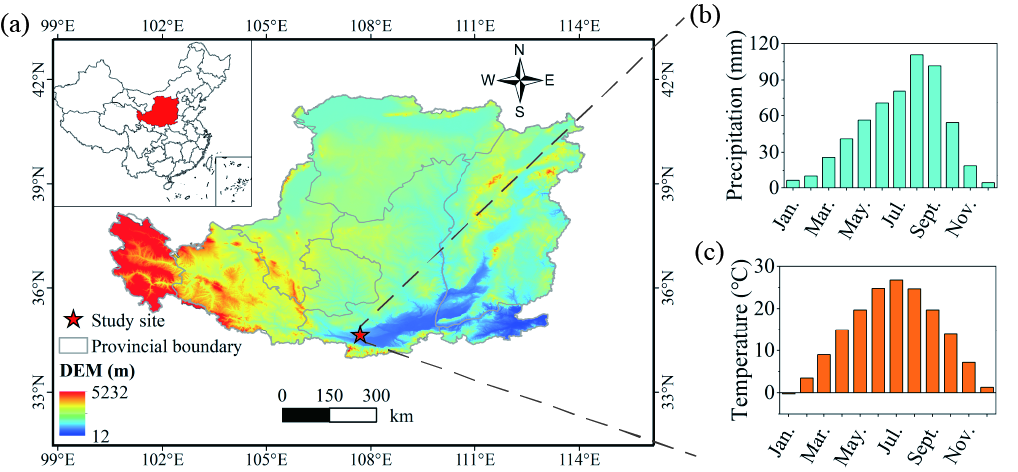


**Figure S1.** Elevation of Loess Plateau in China and location of test points (a); 30-year average monthly precipitation of the test station (b); average monthly daily temperature of the test station for 30 years (c).


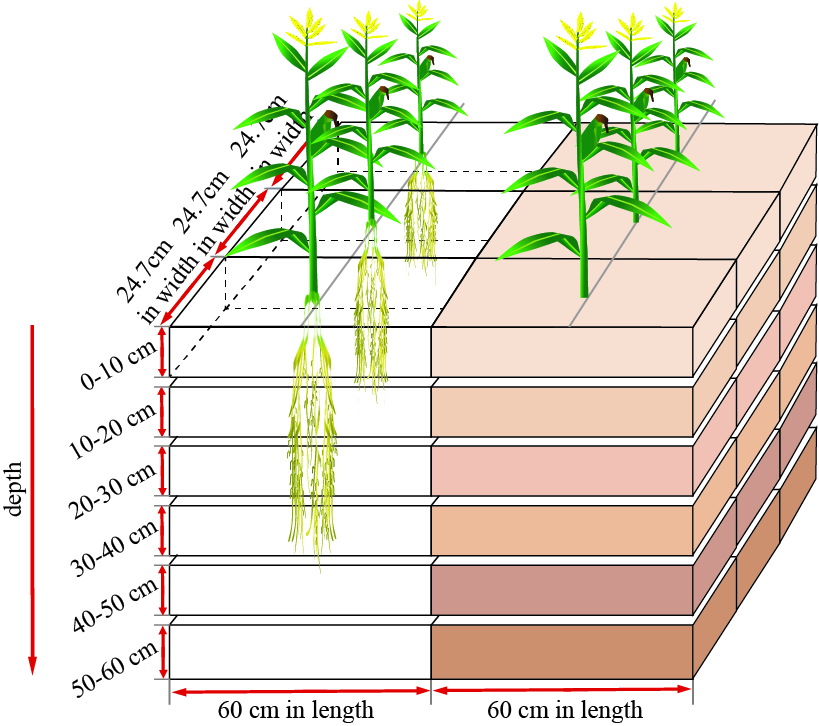


**Figure S2.** Schematic diagram of maize root sampling method.


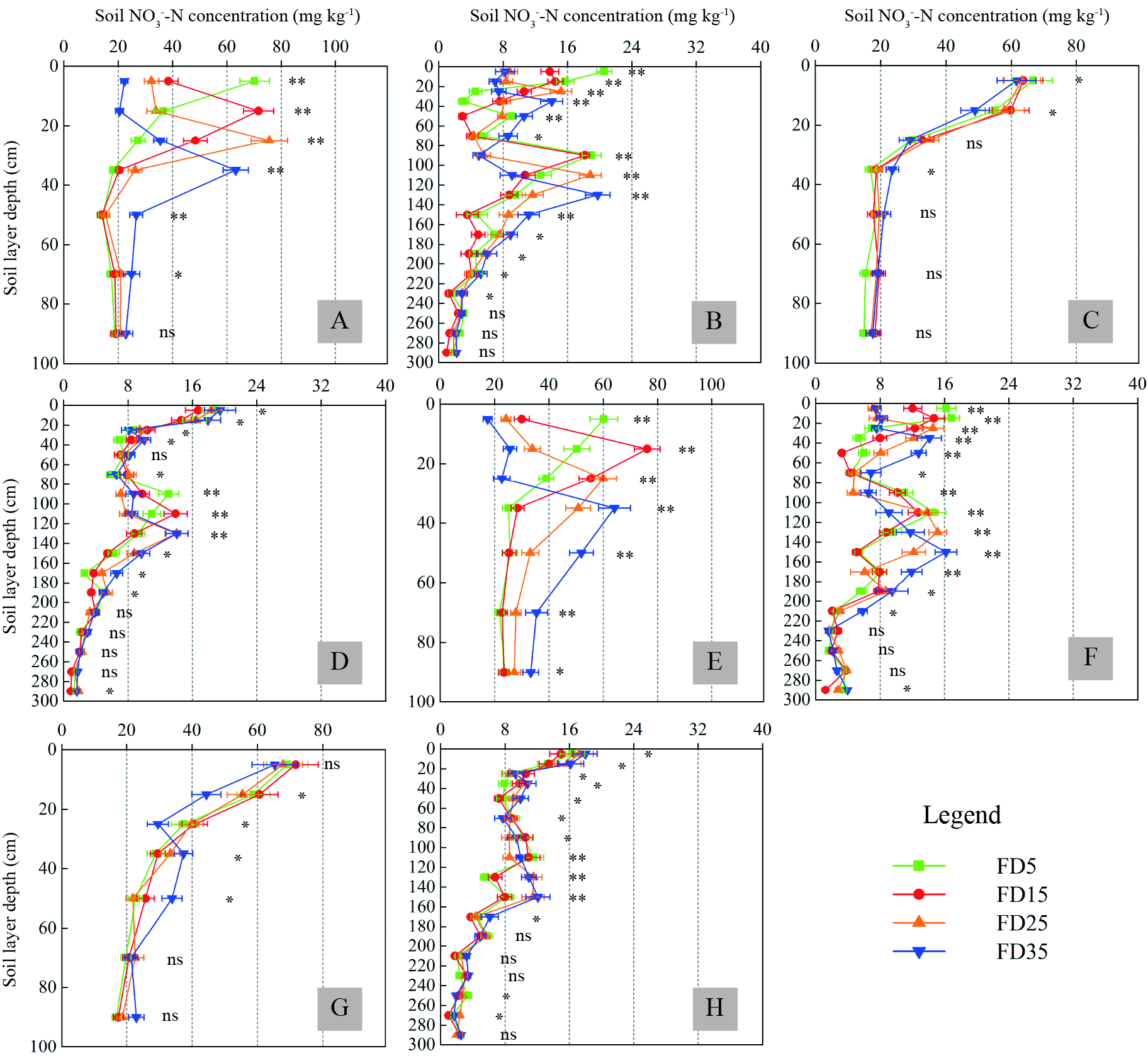


**Figure S3.** Soil nitrate concentration at different sampling times under different fertilizer application depths. Average soil nitrate concentration **(A)** in 2019 summer maize growing season, **(C)** in 2019-2020 winter wheat growing season, **(E)** in 2020 summer maize growing season, and **(G)** in 2019-2020 winter wheat growing season. Soil nitrate concentration **(B)** at the 2019 summer maize maturity, **(D)** at 2019-2020 winter wheat maturity, **(F)** at 2020 summer maize maturity, and **(H)** at 2020-2021 winter wheat maturity. FD5, fertilizer application at a depth of 5 cm; FD15, fertilizer application at a depth of 15 cm; FD25, fertilizer application at a depth of 25 cm; and FD35, fertilizer application at a depth of 35 cm. The vertical bars stand for standard deviation. ns, no significant difference; *, significant at *P* < 0.05; **, significant at *P* < 0.01.


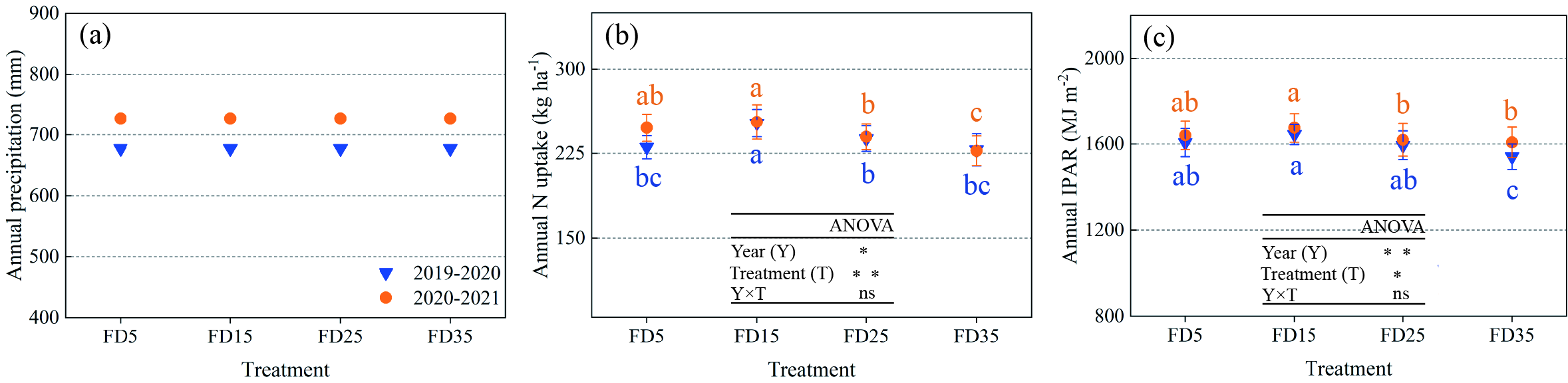


**Figure S4.** Annual precipitation (a), annual N uptake (b) and annual IPAR (c) by summer maize and winter wheat. IPAR, intercepted photosynthetically active radiation; FD5, fertilizer application at a depth of 5 cm; FD15, fertilizer application at a depth of 15 cm; FD25, fertilizer application at a depth of 25 cm; and FD35, fertilizer application at a depth of 35 cm. The vertical bars stand for standard deviation. The different lowercase letters indicate a significant difference among the treatments at *P* < 0.05 using the LSD method. ns, no significant difference; * significant at *P* < 0.05; ** significant at *P* < 0.01.

1. **References in supplementary**

Pickering N B, Hansen J W, Jones J W, et al. 1994. WeatherMan: Autility for managing and generating daily weather data. Agronomy Journal, 86: 332-337. https://doi.org/10.2134/agronj1994.00021962008600020023x
